# Identification of Soybean Genes Whose Expression is Affected by the *Ensifer fredii* HH103 Effector Protein NopP

**DOI:** 10.3390/ijms19113438

**Published:** 2018-11-02

**Authors:** Jinhui Wang, Jieqi Wang, Chunyan Liu, Chao Ma, Changyu Li, Yongqian Zhang, Zhaoming Qi, Rongsheng Zhu, Yan Shi, Jianan Zou, Qingying Li, Jingyi Zhu, Yingnan Wen, Zhijun Sun, Hanxi Liu, Hongwei Jiang, Zhengong Yin, Zhenbang Hu, Qingshan Chen, Xiaoxia Wu, Dawei Xin

**Affiliations:** 1 Key Laboratory of Soybean Biology of Chinese Ministry of Education, Key Laboratory of Soybean Biology and Breeding/Genetics of Chinese Agriculture Ministry, College of Science, Northeast Agricultural University, Harbin 150030, China; jinhuiwang113@126.com (Jin.W.); jieqi0719@126.com (Jie.W.); cyliucn@126.com (Chu.L.); mcneau@163.com (C.M.); m15754506980@163.com (Cha.L.); qian13039802199@163.com (Y.Z.); rshzhu@126.com (R.Z.); llowkeyy@126.com (Y.S.); nachtschatten1994@163.com (Jia.Z.); 15545127190@163.com (Q.L.); jingyizhu1107@126.com (Jin.Z.); wyn8952@126.com (Y.W.); sunzhijunwork@163.com (Z.S.); liuhanxi396525797@163.com (H.L.); j3994102@126.com (H.J.); yinzhengong@163.com (Z.Y.); hzb_net@126.com (Z.H.); 2 Heilongjiang Academy of Agricultural Sciences, Harbin 150030, China

**Keywords:** soybean, NopP, symbiosis, QTL

## Abstract

In some legume–rhizobium symbioses, host specificity is influenced by rhizobial nodulation outer proteins (Nops). However, the genes encoding host proteins that interact with Nops remain unknown. We generated an *Ensifer fredii* HH103 NopP mutant (HH103ΩNopP), and analyzed the nodule number (NN) and nodule dry weight (NDW) of 10 soybean germplasms inoculated with the wild-type *E. fredii* HH103 or the mutant strain. An analysis of recombinant inbred lines (RILs) revealed the quantitative trait loci (QTLs) associated with NopP interactions. A soybean genomic region containing two overlapping QTLs was analyzed in greater detail. A transcriptome analysis and qRT-PCR assay were used to identify candidate genes encoding proteins that interact with NopP. In some germplasms, NopP positively and negatively affected the NN and NDW, while NopP had different effects on NN and NDW in other germplasms. The QTL region in chromosome 12 was further analyzed. The expression patterns of candidate genes *Glyma.12g031200* and *Glyma.12g073000* were determined by qRT-PCR, and were confirmed to be influenced by NopP.

## 1. Introduction

Soybean (*Glycine max (L.) Merr*.), which is an important protein source for humans and animals [1], establishes a symbiotic relationship with rhizobia in different soil environments [2]. Rhizobia can enter soybean roots by infecting root hairs, after which they can stimulate the curling of root hair tips and the formation of an infection chamber containing entrapped bacteria. Rhizobia enters root hairs through infection threads that develop within epidermal root hair cells and migrate to the inner root tissues. The infection thread, containing dividing bacteria, elongates and forms branches to reach root cortical cells where a nodule primordium is formed [3]. When rhizobia (*Bradyrhizobium* and *Ensifer* species) symbiotically colonize soybean roots, the plants can fix atmospheric nitrogen in a process known as biological nitrogen fixation (BNF) [4], which is an efficient and environmentally friendly source of nitrogen. *Ensifer fredii* HH103 strain was first isolated as a fast-growing *Rhizobium japonicum* from a soybean nodule in China [5,6]. Previous studies revealed that *E. fredii* strains grow much faster than *Bradyrhizobium* species, and can nodulate at least 79 different genera of legumes (i.e., a very broad host range) [7,8]. Specifically, *E. fredii* HH103 can nodulate many legumes, including soybean, which is considered its natural host plant [9]. Since genomic and transcriptomic studies of *Ensifer fredii* HH103 are available, this strain is a good candidate for investigating specific gene functions [8,10,11,12,13]. Also, the capacities of *Ensifer fredii* HH103 to grow faster than bradyrhizobia and nodulate soybean, make this strain valuable for understanding molecular mechanisms acting in the soybean-rhizobia interaction.

The nodule organogenesis process requires different signal exchanges between the symbiotic partners [14]. Understanding these signal exchange mechanisms is necessary to apply BNF in agriculture. For example, Nod factors are essential for the initiation of symbiosis with legumes. Meanwhile, flavonoids from host plants interact with rhizobial NodD proteins to activate the production of Nod factors. Legumes perceive Nod factors via Nod-factor receptors, such as MtNFH1, to regulate nodulation. Additionally, the Nod-factor signaling pathway induces the expression of the symbiosis-related genes required for bacterial infections and nodule formation [15]. Rhizobia have many microbe-associated molecular patterns (MAMPs), including flagellin, lipopolysaccharides, exopolysaccharides, and β-glucans, but limited searches indicated that these rhizobial MAMPs appear to lack MAMP activities [16]. One study concluded that a peptidoglycan-modifying enzyme in rhizobia is required for bacteroid differentiation in *Aeschynomene* species, implying that peptidoglycans influence nodule functions [17]. In the legume–rhizobium interaction, effector- or MAMP-triggered plant immunity mediated by host receptors is important for regulating the rhizobial host range [18,19]. Rhizobia can inject many effector proteins into host cells via type III, type IV, or type VI secretion systems. The roles of these effectors are presumably similar to those of effectors from plant pathogens, which promote the infection process [20,21]. The mechanisms underlying signal exchanges will need to be characterized to enhance the application of BNF as an agricultural practice. In some legume–rhizobium symbioses, the type III secretion system (T3SS) is important for nodulation and host-range determination [8,22,23,24]. In rhizobia, the proteins secreted by the T3SS are collectively known as nodulation outer proteins (Nops); at least 11 secreted proteins, NopA, NopB, NopC, NopD, NopJ, NopL, NopM, NopI, NopP, NopT and NopX have been identified in *Ensifer fredii* [25,26,27,28,29,30,31,32,33,34,35,36,37,38,39], all of them being present in strain HH103 [9].

The NopP type III-secreted effector protein was first identified in *R. fredii* (*E. fredii*) strain NGR234 [27]. The *E. fredii* USDA257 NopP is secreted into the interior of cowpea root cells, suggesting it may be a real effector [40]. The NopP identified in *E. fredii* HH103 is similar to that of NGR234, and can influence *GmPR1* expression during the HH103 infection of soybean [41]. Earlier studies determined that GmPR1 is a member of a protein family that includes enzymes (e.g., chitinase and β-1,3-glucanase) that can directly attack pathogen structures, thereby acting as antimicrobial compounds [42,43,44]. Similar to NopL, NopP is also phosphorylated by an unknown kinase in plants, but its specific function is unknown [30]. In plants, NopP may interact with anti-pathogenic signals, like NopL, because NopP is also phosphorylated by its substrate [41]. Additionally, *Rj2* encodes a typical R protein belonging to the Toll-interleukin receptor/NBS/LRR class. Previous studies confirmed that Rj2 restricts the nodulation involving specific rhizobial strains, such as *Bradyrhizobium diazoefficiens* USDA122 [45,46]. The results of the current study indicate that Rj2-producing soybean plants may restrict nodulation via effector-triggered immunity (ETI). The inactivation of the T3SS restores the ability of *B. diazoefficiens* USDA122 to nodulate the Rj2-producing soybean cultivar Hardee [47]. A recent study revealed that NopP interacts with Rj2 to mediate the incompatibility between rhizobia and soybean. However, the NopP effector and the molecular mechanism underlying this genotype-specific incompatibility with the Rj2-producing soybean remain unknown. Many researches have proved that NopP played an important role during rhizobia infecting plant, but beside Rj2, no proteins that directly interacts with NopP were found in the legume host [48].

Quantitative trait locus (QTL) mapping is an ideal method for mapping genomic regions associated with ecologically and evolutionarily important traits. However, there are relatively few reports describing the mapping of soybean genes responsible for nodule traits [49]. Research since the 1950s aimed at detecting genomic loci related to nodulation [50] has led to the identification of several loci, including *rj1*, *rj2*, *rj3*, *rj4*, *rj5*, *rj6*, *rj7*, and *rj8* [51,52,53,54,55]. Moreover, *rj2* and *rj4* were recently cloned [56,57]. The *rj2* gene encodes a determinant of symbiotic specificity that is dependent on the T3SS in USDA257 [58]. Furthermore, *Rj4* reportedly regulates the incompatibility and compatibility between soybean and rhizobia, and type III effectors of *Bradyrhizobium elkanii* may be associated with the incompatibility with soybean carrying the *Rj4* gene [59]. Identification of host genes interacting with specific rhizobial effectors would facilitate the understanding of the molecular mechanisms involved in the host regulation of symbiosis.

In this study, we identified QTLs encoding proteins that interact with NopP in a recombinant inbred line (RIL) derived from a cross between soybean germplasms Dongnong594 and Charleston, which have contrasting nodule phenotypes. Additionally, soybean genes whose expression is affected by NopP were identified.

## 2. Results

### 2.1. NopP Effects on Soybean Germplasm

**Figure 1:**
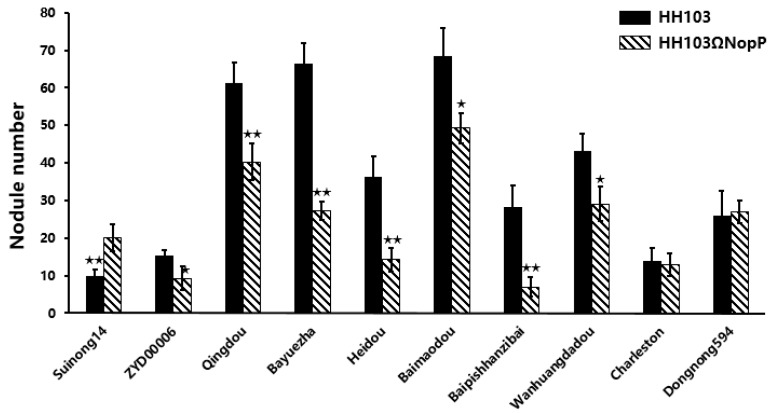

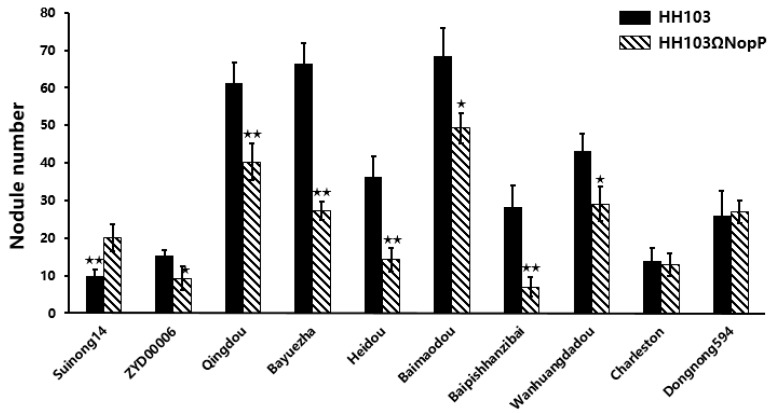
Nodule phenotype of soybean germplasm after inoculation with *Ensifer fredii* HH103 and *Ensifer fredii* HH103ΩNopP. Nodulation tests were performed three times; *t*-test was performed comparing the NopP mutant to the wild type strain; when significant (0.01 ≤ *p* ≤ 0.05), an asterisk is shown, ** indicate *p* ≤ 0.01. Ecoregions origin of germplasms: Suinong14 (Heilongjiang), ZYD00006 (Heilongjiang), Qingdou (Shanxi), Bayuezha (Zhejiang), Heidou (Zhejiang), Baimaodou (Zhejiang), Baipishanzibai (Shanxi), Wanhuangdadou (Anhui), Charleston (America), Dongnong594 (Heilongjiang).

In this study, we used 10 soybean germplasms derived from different ecoregions to elucidate the role of NopP in the symbiotic relationships between rhizobia and soybean. In most soybean germplasms, the nodule number (NN) and nodule dry weight (NDW) were significantly different depending on whether the germplasms were inoculated with the NopP mutant or the wild-type strain. Additionally, NopP had a negative effect on the NNs of germplasms, except for the landraces Suinong14, Qingdou and Dongnong594 (Figure 1). However, Qingdou inoculated with HH103 has around 50% more nodules than Qingdou inoculated with HH103ΩNopP. In Dongnong594, the NDW increased in response to the infection by the *E. fredii* HH103ΩNopP strain. Thus, NopP positively and negatively affected the symbiotic relationships between HH103 and soybean, with the differences in the symbiotic phenotypes among germplasms influenced by their genetic backgrounds.

### 2.2. Phenotypic Analysis

The NN and NDW values were significantly different between Charleston and Dongnong594 (Table 1). The NN and NDW data for the RIL population varied, with no significant trend observed for NN. A comparison of the NDWs for the RILs suggested that the NopP mutant increased the NDW in most RILs.

### 2.3. Mapping of Conditional QTL for Nodulation-Related Traits

**Figure 2:**
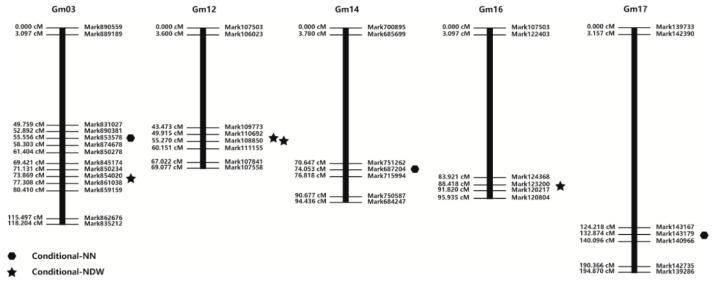
Chromosomal QTL locations of regions associated with the nodule traits of soybean.

Three and four conditional QTLs were identified for NN and NDW, respectively, after inoculations with the wild-type *E. fredii* HH103 and NopP mutant strains (Table 2 and Figure 2). The conditional QTLs for NN were located on Gm03, Gm14, and Gm17, while the conditional QTLs for NDW were located on Gm03 (1), Gm12 (2), and Gm16 (1).

No overlapping QTLs were detected for NN and NDW, although two QTLs located on Gm12 were close to each other, with one at 51.8 cM and the other at 55.5 cM. These two QTLs had not been previously identified, but as previous studies had mapped interaction in some adjacent QTLs with nodulation or pathogen-resistance [49,50], we were interested in whether the QTLs in our study could interact with NopP. As previously mentioned, NopP may be phosphorylated by a host kinase, and it can also influence legume immunity (e.g., ETI). Predicted soybean protein-coding DNA sequences from QTL regions and adjacent regions were retrieved from the Phytozome database (www.phytozome.net/soybean) and then annotated by using them as queries in BLASTX searches of the *Glycine max* Wm82 proteome. On the basis of the main protein domains, we selected 17 genes related to disease-resistance, signal transduction, and nodule formation on Gm12 for further analyses (Table 3).

### 2.4. Validation of Candidate Genes by qRT-PCR

**Figure 3:**
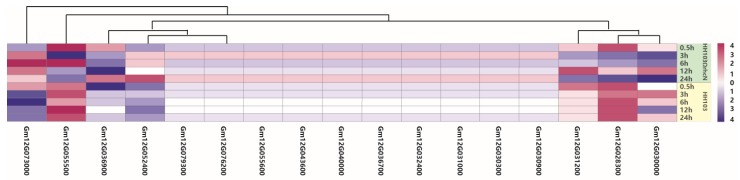
Heat map diagrams of relative expression levels of candidate genes inoculation with *Ensifer fredii* HH103 and *Ensifer fredii* HH103ΩrhcN, 0.5, 3, 6, 12, and 24 h post inoculation. Heatmap was plotted using heatmap.2 function of the R/Bioconductor package gplots. Hierarchical clustering of the DEGs was done by complete method with Euclidean distance. The gene expression levels were transformed by log2 (FPKM + 1) and the values were centered and scaled in row direction. X-axis, differentially expressed gene names; Y-axis, samples. Purple and blue colors indicate a relative increase or decrease in expression inoculation with *Ensifer fredii* HH103 and *Ensifer fredii* HH103ΩrhcN. Seven genes (*Glyma.12g028300*, *Glyma.12g030000*, *Glyma.12g036900, Glyma.12g052400*, *Glyma.12g055500, Glyma.12g031200* and *Glyma.12g073000*) had different expression patterns inoculation with *Ensifer fredii* HH103 comparison with *Ensifer fredii* HH103ΩrhcN mutant.

**Figure 4:**
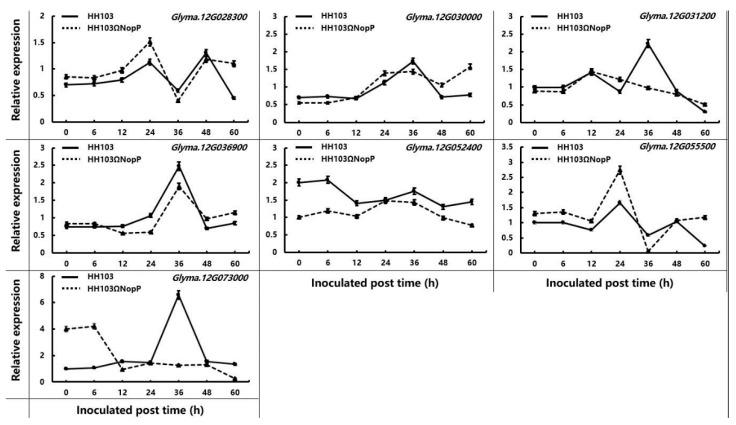
Relative expression levels of *Glyma.12g028300*, *Glyma.12g030000*, *Glyma.12g031200*, *Glyma.12g036900*, *Glyma.12g052400*, *Glyma.12g055500* and *Glyma.12g073000* in soybean roots after being independently inoculated with *Ensifer fredii* HH103 and *Ensifer fredii* HH103ΩNopP in ‘Charleston’, and non-inoculated plants as the control. The expression levels of target genes relative to the control samples at corresponding time points were calculated using the 2^−ΔΔ*C*t^ method. Bars represent the mean ± SE (standard error) of three replications. Different letters represent significant differences (*p* ≤ 0.05) at each time point based on Duncan’s multiple range test.

We analyzed the transcriptome of soybean germplasm Suinong14 inoculated with *E. fredii* HH103 or the rhcN mutant strain (data unpublished). The HH103 *rhcN* gene encodes a protein that is 99.56% similar to the NGR234 rhcN protein, which is a T3SS protein that is similar to an ATPase believed to activate the secretion machinery and whose mutation eliminates T3SS secretion [60]. The *rhcN* gene was disrupted with a kanamycin-resistance cassette to generate the HH103ΩrhcN mutant. Subsequent inoculation experiments revealed that the wild-type HH103 and mutant HH103ΩrhcN *E. fredii* strains induced different expression patterns for seven genes (*Glyma.12g028300*, *Glyma.12g030000*, *Glyma.12g036900*, *Glyma.12g052400*, *Glyma.12g055500*, *Glyma.12g031200*, and *Glyma.12g073000*) (Figure 3 and Table 4). The transcriptome analysis suggested that these seven genes may affect T3SS signaling during rhizobial infections. These results implied that the expression of these genes was induced by rhizobia. However, the nodule phenotype differed between Sunong14 and Charleston. To determine whether these seven genes were associated with NopP, Charleston plants inoculated with *E. fredii* HH103 or HH103ΩNopP were analyzed in a qRT-PCR assay, with non-inoculated plants as controls (Figure 4). Of these genes, only *Glyma.12g031200* and *Glyma.12g073000* were differentially expressed between Charleston plants inoculated with the wild-type or mutant *E. fredii* strains. Additionally, *Glyma.12g031200* and *Glyma.12g073000* were similarly expressed in Charleston plants regardless of the *E. fredii* strain. The peak *Glyma.12g031200* and *Glyma.12g073000* expression levels induced by the HH103 strain were detected at 36 h after inoculations, after which the expression levels decreased. In contrast, the HH103ΩNopP strain had no effects on the expression patterns of these two genes. At 36 h after inoculations, the *Glyma.12g031200* expression level was 2.3-times higher in plants inoculated with HH103 than in plants inoculated with HH103ΩNopP, while the *Glyma.12g073000* expression level was 5.4-times higher in plants inoculated with HH103 than in plants inoculated with HH103ΩNopP. The qRT-PCR data supported the hypothesis that Glyma.12g031200 and Glyma.12g073000 interact with NopP.

## 3. Discussion

Although several QTLs were detected in this study, it was difficult to determine which were associated with NopP. Nodule development in legumes (e.g., soybean) involves many processes. The detection of multiple QTLs may be indicative of the complexity in the signal exchange required between rhizobia and hosts to establish symbiosis. A previous study revealed that the correlation coefficients between BNF activity from acetylene reduction assays and NN and NDW were 0.44 and 0.70, respectively [49]. The locus on Gm03 detected in the current study overlaps a previously identified QTL related to NDW [61]. Additionally, a locus related to NN on Gm16 [61] overlaps a locus adjacent to a QTL we detected. The conditional QTLs associated with NopP may be useful for identifying genes encoding proteins that interact with NopP. In this study, the detection of overlapping loci on Gm12 implies that the region encodes an interacting partner of NopP. Therefore, we analyzed the loci on Gm12 in greater detail.

The transcriptome analysis and qRT-PCR results indicated that the expression of two genes (*Glyma.12g031200* and *Glyma.12g073000*) was interesting: They had a similar expression pattern after inoculation with the parental strain (maximum at 36 h post inoculation) that was not observed after inoculation with HH103ΩNopP. This fact suggests that these two genes might be involved in the NopP-triggered signal pathway during nodule organogenesis. The *Glyma.12g031200* encoded protein belongs to the pathogenesis-related 5 (PR5) family and shows amino acid sequence and protein structural similarities to the sweet tasting protein from *Thaumatococcus daniellii* [62], also named thaumatin-like proteins (TLPs). Many PR5 proteins have been identified in a wide range of plant species, such as wheat [63], soybean [57] and *Avena sativa* [64]. Some PR5 proteins have been proposed to participate in resistance to some pathogens, such as *Arabidopsis thaliana ATLP3*, that is induced by pathogenic fungi [65]. RlemTLP, a PR5 protein from rough lemon, has also been identified as a protein acting against pathogens [66]. *Rj4*, which codes for a thaumatin-like protein, participates in control nodulation specificity in soybean [57]. The action of Rj4 depends on the rhizobial type III secretion system (T3SS) [67]. The encoded product of *Glyma.12g031200* belongs to the same TLP family that Rj4 and our transcriptome and qRT-PCR results support that NopP could induce *Glyma.12g031200* expression. The fact that *Glyma.12g031200* is not an R gene but encodes a thaumatin-like protein is kind of surprising. It will be interesting to understand how a thaumatin-like protein is involved in a NopP-triggered process in soybean-rhizobia interaction. However, more work is required to confirm the interaction pattern between NopP and *Glyma.12g031200*.

*Glyma.12g073000* codes for a mitogen-activated protein kinase 3 (MAPK3), an important member of the MAPKs cascade. The mitogen-activated protein kinase (MAPK) genes are involved in various signalling pathways associated with biotic and abiotic stress responses in plants [68]. Previous studies have shown that various kinase activities are necessary in root nodule development in legumes [69,70]. *TDY1*, which encodes a MAPKs protein, has been identified to be involved in root nodulation and root tip development in *Medicago* [71]. In another legume, *Lupinus albus*, activation of MAPKs SIMK and SAMK is required for infection by bradyrhizobia [72]. Another study showed that a gene called *GMK1*, believed to encode a MAPK homolog, is involved in the symbiotic interaction between *Bradyrhizobium japonicum* USDA110 and soybean [73]. T3SS in bacteria also can mediate MAPKs when microbe infecting cells. For example, VopA, a T3SS effector of *Vibrio parahaemolyticus* could inhibit MAPKs pathway during host infection [74]. In this study, we show that the soybean MAPK3 encoding gene, *Glyma.12g073000*, is induced by HH103 when NopP is present, suggesting that this effector can induce a MAPK cascade in soybean roots. Further research is required to completely elucidate this issue.

The NopP type III effector is conserved in many rhizobial strains, but its effects on nodulation vary depending on the host plant species. For example, during infections of *Pachyrhizus tuberosus*, the *E. fredii* NGR234 mutant lacking a functional NopP cannot nodulate the host plant roots. In contrast, the inactivation of NopP increases the NN in the tropical legumes *Flemingia congesta* and *Tephrosia vogelii* [27]. Although NopP is produced by diverse rhizobia, there is no homology between the genes encoding NopP and Avr proteins from pathogens, suggesting that NopP is an effector that evolved in a legume–rhizobium symbiosis system. Moreover, depending on the NopP variant that interacts with Rj2, certain bradyrhizobial infections may be prevented via ETI [48]. Specifically, ETI is an accelerated and amplified pathogen-associated molecular pattern-triggered immunity response in which an R protein in the host plant directly or indirectly recognizes pathogen effectors, ultimately resulting in disease resistance [75,76]. The expression of many salicylic acid-dependent defense-signaling marker genes (e.g., *PR-1*, *PR-2*, and *PR-5*) may be induced after ETI is activated [77,78]. In soybean, *GmPR1* expression may be upregulated following an infection by *E. fredii* HH103. Furthermore, *PR-2* expression is reportedly significantly upregulated at 2 days after inoculation with *B. diazoefficiens* USDA122 relative to the expression levels in plants inoculated with the USDA122NopP_110_ mutant and in uninoculated controls, but is markedly downregulated at 4 days after inoculation [48]. However, *PR-1* and *PR-5* expression levels are not significantly affected by *B. diazoefficiens* USDA122. The expression of *Glyma.12g031200*, which encodes a PR-5 protein, may be induced by NopP during the HH103 infection process. Thus, *Glyma.12g031200* may be a new PR-5 protein that differs from those previously reported, and it may be involved in the induction of ETI resulting from interactions between NopP and Rj2. Like NopL, NopP can be phosphorylated by plant host kinases [30,38]. The phosphorylation of NopP may influence nodulation by inducing more signaling events. Therefore, the effects of HH103-secreted NopP on symbiotic relationships may be due to the complex interactions between effectors and the host plant. The MAPK-associated pathways have important roles in these complex interactions, and *Glyma.12g073000* (MAPK3) may affect signal transduction pathways during NopP phosphorylation or the ETI-mediated by Rj2.

We propose that interactions between phosphorylated NopP and Rj2 in legumes induce signaling that upregulates *TLP* and *MAPK3* expression to regulate nodule organogenesis. In this study, *Glyma.12g031200* and *Glyma.12g073000* were mapped using QTLs, and may be useful for detecting NopP signals involved in specific plant host signaling pathways. Identifying the genes encoding proteins that interact with specific type III effectors will increase our understanding of how symbiosis is established between rhizobia and soybean.

## 4. Materials and Methods

### 4.1. Strains, Plasmids and Primers

*Ensifer fredii* HH103 and its derived mutants, as well as *Escherichia coli* ‘DH5α’, were used in this study. Primers and plasmids are listed in Tables S1 and S2.

### 4.2. Generation of the HH103ΩNopP Mutant

To generate the *E. fredii* HH103ΩNopP mutant, a 1.8-kb fragment containing *NopP* (GenBank: AAY33495.1) was cloned into pGWC to construct the pGWC-*NopP1800* vector. A *BamH*I restriction enzyme site was introduced close to the ATG codon of *NopP* by PCR-based site-directed mutagenesis. An Ω interposon with the kanamycin resistance gene was excised from pEASY-T1 (Transgene Biotech Co., Beijing, China) by a PCR amplification with gene-specific primers with *BamH*I restriction enzyme sites (Table S1). The amplified fragment was purified, digested with *BamH*I, and ligated into the *BamH*I-digested pGWC-*NopP1800* to generate pGWC-*NopP2800Ω*. The construct was then cloned into the suicide vector pJQ200SK [79]. The resulting plasmid, pJQ-*NopP2800Ω*, was mobilized from *E. coli* (DH5α) cells into *E. fredii* HH103 by triparental mating with the pRK2013 helper plasmid [80]. Putative transformants were detected based on kanamycin resistance and growth on 5% (*w*/*v*) sucrose. The obtained *E. fredii* HH103ΩNopP mutant was confirmed by a Southern blot analysis with a DIG DNA-labeling and detection kit (Roche, Basel, Switzerland).

### 4.3. Nodulation Tests

#### Plant Materials and Nodulation-Related Traits

The germplasms used were Baimaodou, Qingdou, Huangpishanzibai, Wanhuangdadou, Heidou, Bayuezha, Suinong14, ZYD00006, Dongnong594 and Charleston, which are derived from different ecoregions. All of the seeds of the different populations and germplasms were surface sterilized and germinated on sterile plastic plates. Plantlets were transferred to 300-mL plastic jar (Magenta jar) units linked with a cotton wick (a mixture of vermiculite and expanded clay in the upper vessel; nitrogen-free nutrient solution in the lower vessel) under greenhouse conditions [7,30]. Five replicates and three independent experiments were performed. The soybean seeds were subjected to three major seed sterilization treatments, using chlorine gas, commercial Clorox bleach, containing 5.25% bleach, and 3% (*v*/*v*) hydrogen peroxide, and were then germinated on agar plates. Plants (one plant per jar) were inoculated with 109 bacteria (strain HH103 and mutant derivatives; see Table S2) at the Vc stage [61]. Plants were cultivated at 25 ± 2 °C in a temperature-controlled greenhouse with a 16-h photoperiod. Four weeks after inoculation, plants were harvested for nodulation evaluation, as assessed by nodule number (NN) and nodule dry weight (NDW). *t*-tests were used to detect the statistical significances of phenotypes.

### 4.4. The Conditional QTL Mapping of Nodulation-Related Traits

The experimental RIL population used in this study (‘Charleston’ × ‘Dongnong594′, *n* = 150) originated from two individual lines, Charleston and Dongnong594 [81]. To map QTLs controlling nodule-related traits in the RIL population of ‘Charleston’ × ‘Dongnong594′, we used the method of composite interval mapping [61], with WinQTL Cartographer [61]. The control marker number and window size were five and 10 cM, respectively. A walk speed of 0.5 cM and the forward regression method were selected. The proportion of the variance explained by each particular QTL and the additive effects were obtained from the composite interval mapping analysis. LOD score peaks greater than 2.0 (WinQTL Cartographer default threshold) indicated the existence of conditional QTLs for the two nodule traits after being independently inoculated with the two type strains studied here. For the additive-effects signals, ‘+’ indicates increasing allelic effects from ‘Dongnong594′ and ‘−’ indicates decreasing allelic effects from ‘Charleston’. The experimental threshold levels for linkage were calculated from 1000 permutations of each genotypic marker against the phenotype in the population. Linkage was reported as significant if the two values for a marker were greater than the critical value at *p* = 0.05 [82]. The *NopP* genes results in differences between the parental strains and *Ensifer fredii* ‘HH103ΩNopP’. The differences in the phenotypic values for the two strains was caused by NopP. The differences in phenotypic values were used to determine the conditional QTLs’ locations [61].

### 4.5. Delimitation of QTL Regions and Identification of Candidate Genes

The identification of genes within the QTL regions was performed as described previously [1]. Briefly, each QTL was bound by two specific-length amplified fragment markers from a previously published high-quality genetic map [83]. A BLAST algorithm-based search of the soybean genome was performed (http://soybase.org/gbrowse/cgi-bin/gbrowse/gmax1.01/), and gene names and descriptions were obtained (http://soybase.org/gbrowse/cgi-bin/gbrowse/gmax1.01/) [61]. The predicted coding DNA sequences for QTL regions in soybean were retrieved from the Phytozome website (www.phytozome.net/soybean) and were annotated by querying them against the *G. max* ‘Wm82′ proteome using BLASTX [82].

### 4.6. RNA Isolation and qRT-PCR Analyses of NopP Candidate Genes

To identify candidate genes that may interact with NopP, qRT-PCR was performed to measure the relative transcript levels of these genes in ‘Charleston’. The total RNAs of the roots were isolated using an EasyPure^®^ Plant RNA Kit (Transgene Biotech Co.), followed by an RNase-free gDNA wiper treatment (Vazyme Biotech Co., Nanjing, China), and then each RNA sample was converted into cDNA using the HiScript^®^ II Q RT SuperMix (Vazyme Biotech Co.). Root samples were taken at 0, 12, 24, 36, 48 and 60 h after independent inoculations with *Ensifer fredii* ‘HH103ΩNopP’ and the relative parental strain. They were ground to a fine powder in a liquid nitrogen-precooled mortar. qRT-PCR was performed with ChamQ™ Universal SYBR^®^ qPCR Master Mix (Vazyme Biotech Co.) on a Roche LightCycler 480 II System (Roche). The qRT-PCR program was as follows: denaturation at 95 °C for 2 min, followed by 40 cycles of 95 °C for 10 s, 60 °C for 30 s and 72 °C for 30 s. All RNA extractions were performed in three biological replicates, and each cDNA sample was analyzed three times. *GmUNK1* (*Glyma.12g020500*) was used as the reference gene to calibrate the transcript abundance values among different samples [84]. The Ct values were calculated by the Roche LightCycler 480 II software. The primer sequences of the qRT-PCR target genes are listed in Table S1.

## Figures and Tables

**Table 1:** Parental and population statistics for nodule traits in the soybean ‘Charleston’ × ‘Dongnong594’ population.

	RILs (*n* = 150)				Parents (Average)	
	Traits	Average	Standard Deviation	Coefficient of Variation	Charleston	Dongnong594
HH103 Rif^R^	Nodule number	23.9	15.1	76.38	14.0 ± 3.5 **	26.0 ± 6.6
	Nodule dry-weight (mg)	18.4 *	17.2	93.25	13.0 ± 2.6 **	22.5 ± 1.8
HH103 Rif^R^ΩNopP	Nodule number	11.5 **	13.822	58.07	13.0 ± 3.0 **	27.0 ± 3.0
	Nodule dry-weight (mg)	29.4	0.0236	80.34	10.0 ± 2.6 **	41.5 ± 5.6

* indicate *p* ≤ 0.05, ** indicate *p* ≤ 0.01.

**Table 2:** Chromosomal localization of conditional QTLs for nodule-related traits.

Trait	LG/QTL	Chrom.	Position (cM)	LOD ^a^	R^2^ (%) ^b^	ADD ^c^
Nodule number	QN/NN01	03	55.5	3.8	8.21	−2.46
	QB2/NN02	14	74.0	4.6	9.04	−2.95
	QD2/NN03	17	133.2	5.0	8.19	2.61
Nodule dry weight	QN/NDW01	03	74.0	5.0	2.54	−0.001
	QH/NDW02	12	51.8	4.0	9.78	0.005
	QH/NDW03	12	55.5	5.4	3.75	0.002
	QJ/NDW04	16	88.8	4.5	5.26	−0.002

^a^ LOD: log of odds. ^b^ R^2^ (%): the contribution rate of the QTL; ^c^ ADD: the additive effects contributed by the QTL; LG: Linkage group; QN: QTL on LG N, QB2: QTL on LG B2, QD2: QTL on LG D2, QH: QTL on LG H, QJ: QTL on LG J.

**Table 3:** Relevant information of candidate genes belonging to the two overlapping conditional QTLs for NDW.

No.	Gene	Function
1	*Glyma.12G028300*	Cell elongation protein/DWARF1/diminuto (DIM)
2	*Glyma.12G030000*	Leucine-rich repeat protein kinase family protein
3	*Glyma.12G030300*	Disease resistance-responsive (dirigent-like protein) family protein
4	*Glyma.12G030900*	Like auxin resistant 2
5	*Glyma.12G031000*	Pathogenesis-related thaumatin superfamily protein
6	*Glyma.12G031200*	Pathogenesis-related thaumatin superfamily protein
7	*Glyma.12G032400*	Calcium-dependent lipid-binding (CaLB domain) family protein
8	*Glyma.12G036700*	NAD(P)-linked oxidoreductase superfamily protein
9	*Glyma.12G036900*	NAD(P)-binding rossmann-fold superfamily protein
10	*Glyma.12G040000*	Leucine-rich receptor-like protein kinase family protein
11	*Glyma.12G043600*	Protein kinase superfamily protein
12	*Glyma.12G052400*	Protein kinase family protein
13	*Glyma.12G055500*	Leucine-rich repeat (LRR) family protein
14	*Glyma.12G055600*	Leucine-rich repeat (LRR) family protein
15	*Glyma.12G073000*	Mitogen-activated protein kinase 3
16	*Glyma.12G076200*	Auxin response factor 10
17	*Glyma.12G079300*	SAUR-like auxin-responsive protein family

**Table 4:** Annotation of candidate genes might interact with NopP.

No.	Gene	Function
1	*Glyma.12G028300*	Cell elongation protein/DWARF1/Diminuto (DIM)
2	*Glyma.12G030000*	Leucine-rich repeat protein kinase family protein
3	*Glyma.12G031200*	Pathogenesis-related thaumatin superfamily protein
4	*Glyma.12G036900*	NAD(P)-binding rossmann-fold superfamily protein
5	*Glyma.12G052400*	Protein kinase family protein
6	*Glyma.12G055500*	Leucine-rich repeat (LRR) family protein
7	*Glyma.12G073000*	Mitogen-activated protein kinase 3

Gene name and the functional annotation depending on the genomic information of soybean in phytotomy (https://phytozome.jgi.doe.gov/pz/portal.html#!info?alias=Org_Gmax).

## Supplementary Materials

The following are available online at http://www.mdpi.com/1422-0067/19/11/3438/s1, Table S1, Information of Primers Used; Table S2. Information of Strains and Vectors.
