# Supplementary material for: Identification of Soybean Genes Whose Expression is Affected by the Ensifer fredii HH103 Effector Protein NopP
Source: Int J Mol Sci. 2018 Nov 2;19(11):3438. doi: 10.3390/ijms19113438 (PMC6274870; doi:10.3390/ijms19113438)
Supplement: Supplementary file 1 [file ijms-19-03438-s001.pdf]

Table S1. Information of Primers Used.

No.	Name of primer	Primer 5′-3′	Annotation and Functions
1	pGWC-*NopP*1.8-F-	CTTCAGATATGTTTCGCGAGG	Clone the fragment containing *NopP* and putative promoter into pGWC
2	pGWC-*NopP*1.8-R-	AACACCGAATGGGTATCGCTC	Clone the fragment containing *NopP* and putative promoter into pGWC
3	*NopP* Ω*Bam*H I F	TCGCCATGTACGGTCGGATCCATAGCTCCGATT	Site-directed mutagenesis, mutated *NopP*1.8 had a *BamH1* restriction site
4	*NopP* Ω*Bam*H I R	AGCGGTTGCCAGCCTAGGTATCGAGCAGGCTAA	Site-directed mutagenesis
5	Kan-*BamH1*-F	tcaagtAGTAAACTGGATGGCTTTCTTG	Clone Kanamycin fragment into pGWC-*NopP*1.8
6	Kan-*BamH1*-R	tcaagtCTTCAGCATCTTTTACTTTCAC	Clone Kanamycin fragment into pGWC-*NopP*1.8
7	pJQ200SK-*NopP*Ω-F	GCTCTAGACTTCAGATATGTTTCGCGAGG	Construction of Suicide vector pJQ200SK-*NopP*Ω to generate HH103Ω*NopP*
8	pJQ200SK-*NopP*Ω -R	AACTGCAGAACACCGAATGGGTATCGCTC	Construction of Suicide vector pJQ200SK-*NopP*Ω to generate HH103Ω*NopP*
9	*GmUNK1*	Fwd-TGGTGCTGCCGCTATTTACTG<br>Rev- GGTGGAAGGAACTGCTAACAAT	qRT-PCR for validation of *GmUKN1* gene transcription level in Charleston
10	*Glyma.12G028300*	Fwd- CTTCGGATAGCCTCAGTTG<br>Rev- AACAACCCGTGAAAGGATT	qRT-PCR for validation of *Gm19g138300* gene transcription level in Charleston
11	*Glyma.12G030000*	Fwd- ATTGGTTATTGATACGATTC<br>Rev- AAAAAGAAAATACAAGACAAAGC	qRT-PCR for validation of *Gm19g140100* gene transcription level in Charleston
12	*Glyma.12G031200*	Fwd- ATTAGTATTATCATCTTGGGAGC<br>Rev- CAACACAGGCACACAGCAC	qRT-PCR for validation of *Gm19g131900* gene transcription level in Charleston
13	*Glyma.12G036900*	Fwd- TTGAAGATGTGGTTGAATTG<br>Rev- TTGGGAATGAAAAGGAAAAT	qRT-PCR for validation of *Gm06G181200* gene transcription level in Charleston
14	*Glyma.12G052400*	Fwd- CTTTTTTCGCCCCCTACTT<br>Rev- GCACCACAACGAAGAGAATG	qRT-PCR for validation of *Gm06G171700* gene transcription level in Charleston
15	*Glyma.12G055500*	Fwd- ATGTTAAGATGTGCCTGAAT<br>Rev- CGTTACAGTCAGAAATCCATAG	qRT-PCR for validation of *Gm19g117200* gene transcription level in Charleston
16	*Glyma.12G073000*	Fwd- ATATTTTTTTTAACCCTGACGG<br>Rev- ATGTTTGTTTTATTTAGTCCCTC	qRT-PCR for validation of *Gm19g129200* gene transcription level in Charleston

Table S2. Information of Strains and Vectors.

Strain	Relevant Characteristics	Reference
Escherichia coli		
DH5α	supE44 lacY169 (80lacZM15) hsdR17 recA1 endA1 gyrA96 thi-1 relA1	Mason et al. 1989
BL21(DE3)	F- ompT hsdSB (rB- mB-) gal dcm (DE3)	Studier et al. 1990
***Rhizobium* strains**		
HH103	Broad host range bacterium isolated from nodules of Glycine max , Rif^r	Dowdle et al. 1985
HH103Ω*NopP*	HH103 insertion mutated containing an Kanamycin resistance gene insertion at position downstream 8bp of start codon of *NopP* nucleotide sequence, Rif^r, Kan^r	This work
**Plasmids**		
pGWC	Entry clone vector,Cm^r	Chen et al. 2006
pJQ200SK	Suicide vector used for directed mutagenesis (Gm^r)	Quandt and Hynes 1993
pJQ200SK-*NopP*Ω	A 2.8kb Xba1-Pst1 fragment containing *NopP* with a Kanamycin resistance gene inserted into downstream 8bp of start codon of *NopP* the Xba1-PstI site of pJQ200SK (Gm^r)	This work
pRK2013	Tra^+ helper plasmid for mobilisation (Kan^r)	Figurski and Helinski, 1979

Note: Rifampicin(Rif^r); Kanamycin (Kan^r); Chloramphenicol (Cm^r); Gentamicin (Gm^r).
